# Inventory Control System for a Healthcare Apparel Service Centre with Stockout Risk: A Case Analysis

**DOI:** 10.1155/2017/9210532

**Published:** 2017-11-19

**Authors:** An Pan, Chi-Leung Hui

**Affiliations:** ^1^The Shanghai Lixin University of Accounting and Finance, 995 Shangchuan Road, Pudong New Area, Shanghai 201209, China; ^2^The Hong Kong Polytechnic University, Hung Hom, Kowloon, Hong Kong

## Abstract

Based on the real-world inventory control problem of a capacitated healthcare apparel service centre in Hong Kong which provides tailor-made apparel-making services for the elderly and disabled people, this paper studies a partial backordered continuous review inventory control problem in which the product demand follows a Poisson process with a constant lead time. The system is controlled by an (*Q*,*r*) inventory policy which incorporate the stockout risk, storage capacity, and partial backlog. The healthcare apparel service centre, under the capacity constraint, aims to minimize the inventory cost and achieving a low stockout risk. To address this challenge, an optimization problem is constructed. A real case-based data analysis is conducted, and the result shows that the expected total cost on an order cycle is reduced substantially at around 20% with our proposed optimal inventory control policy. An extensive sensitivity analysis is conducted to generate additional insights.

## 1. Introduction

This paper reports an analytical study which is based on a real case of a healthcare apparel service centre, called the Troels H. Povlsen Care Apparel Centre (supported by The Hong Kong Polytechnic University). This healthcare apparel service centre is a nonprofit-making organization which provides tailor-made apparel-making services for the elderly and disabled people. With its very small-sized inventory capacity, this service centre cannot keep a lot of fabric materials in stock. Unlike many other apparel providers, the clients of this healthcare centre have special demand on cutting and many of them are willing to wait for some time even when the orders are backlogged (because they cannot find any other organizations which can provide this service with an affordable price). However, backlog and hence “stockout” is a serious problem because the clients do urgently need the apparel products to help them with their living. As a result, the centre tries to achieve a very low stockout level which is termed as “stockout risk.” However, as the centre is nonprofit-making, having an efficient and sustainable operation [1] means the centre also has to minimize the cost.

Concerning such a situation, we study in this paper a single item partially backordered inventory system governed by a continuous review (*Q,r*) policy: (i) when the inventory position (stock on hand plus stock on order minus backorders) reaches the reorder point *r*, an order is placed with the batch size *Q*; (ii) the storage space is capacitated, and unmet demands are partially backordered; and (iii) there is a “free waiting time” during which there is no backorder cost for the model. The optimization problem is to achieve the stockout risk target with the optimal cost minimizing (*Q*,*r*) inventory policy.

Notice that for stochastic inventory control systems, the (*Q*,*r*) policy is one of the most widely used policies. However, it is well-reported that there is no simple solution scheme for computing the optimal parameters of the (*Q*,*r*) policy [2]. To make the situation more challenging, in addition to the target stockout risk consideration, in the healthcare apparel centre's inventory control problem, there is a capacity constraint. Last but not least, facing the stockout risk, some of the demands are lost while some are backlogged because some customers are willing to wait but not all. This situation is known as “partial backlog” in inventory control, and it affects the design of the optimal inventory (*Q*,*r*) policy significantly. Obviously, the conventional (*Q,r*) policies which do not consider stockout risk, capacity, and partial backlog together will fail to be optimal and hence cannot be used to solve the inventory control problem faced by the healthcare apparel service centre mentioned above.

Based on the healthcare apparel service centre's inventory control challenges, this paper aims to develop a novel optimal (*Q*,*r*) policy which can incorporate the stockout risk, storage capacity, and partial backlog into the optimization model. To the best of our knowledge, this optimization problem has not been studied in the literature. In addition, this paper is based on the real case of a healthcare apparel service centre and we also conduct our analysis by using real-world data from the healthcare apparel service (HAS) centre. This is hence a practice-based study with risk considerations. These highlight the novelty of this study and its contributions.

The rest of the paper is organized as follows. In Section 2, we concisely review the related literature on (*Q*,*r*) inventory models and the importance of risk analysis in healthcare services.  Section 3 presents the model, describes the notation, discusses the optimization problems, and provides the algorithm for identifying the optimal solution.  Section 4 gives numerical studies and discusses a few important insights. Concluding remarks are given in Section 5.

## 2. Literature Review

In the literature, different kinds of stochastic inventory control systems are proposed and explored (see [3–9] and [10, 11]). The (*Q,r*)-based optimal inventory control policy is one of them and has been popularly examined. For example, relatively recently, Song et al. [12] discuss the effect of lead time and uncertain demand on the optimal (*Q*,*r*) policy. Berk and Gürler [13] show that a continuous review (*Q*,*r*) policy is reasonably good for a perishable inventory system with fixed shelf lives and study the operating characteristics of the system. Some other studies have conducted analysis and cost evaluation on the continuous review (*Q*,*r*) policy for identical as well as nonidentical retailers (see, e.g., [14–17]). However, the (*Q*,*r*)-related studies reviewed above have not considered many important factors such as capacity (i.e., space limitation), service level, and partial backlog together, which is what this paper aims to address (Studies have been conducted on trade-offs between the service level and inventory (see [18, 19]). In Liu et al. [20], an efficient procedure is presented to minimize the overall inventory for a class of manufacturing and supply systems with each adopting base stock policy while meeting the required service level. However, these papers mainly focus on service level alone but not other factors together. Thus, this paper is different from them.). Thus, from this sense, this paper is addressing a more challenging and general problem than the previous studies.

The other important feature of our model is that we take characteristics of the customers who are willing to accept a reasonable waiting time (Note that the concept of “free waiting time” of some customers is related to the advance demand information (ADI) literature (see [21]). Most previous studies on ADI concentrate on the value of ADI in production-inventory systems, for example, Buzacott and Shanthikumar [18] present a detailed analysis of a single-stage make-to-stock queue with ADI. Wang et al. [22] study inventory management with a service level constraint under a flexible time-window fulfillment scheme. They use an (*s*,*S*) policy and develop algorithms to find the optimal parameters. We consider a continuous review environment and allow for free waiting time in this paper.) after placing their order into account and formulate the inventory control problem as a “partial backordering optimal inventory control problem.” Observe that quite many prior studies have explored the partial backorder issue in inventory management. For instance, Montgomery et al. [23] introduce a partial backorder inventory policy in which a fraction of unfilled demand is backlogged. Kim and Park [24] explore a similar problem and suggest a modified scenario in which the cost of backorder is assumed to be proportional to length of waiting time. Moinzadeh [25] sets customer's waiting time to a constant number, which is similar to us. However, the basic inventory system they adopted is the (*s*-1,*s*) system, and the optimization constraints are totally different. Rabinowitz et al. [26] analyze a (*Q*,*r*) system with an upper bound on backorder. They consider the scenario in which an emergency order will be placed if the number of accumulated backorders is more than the bound. Hu et al. [27] consider a partial backorder inventory problem under a waiting time-dependent backlogging setting.

Another topic relevant to our research is the “quoted service time” issue, which has been commonly studied in queuing theory-related studies. To our knowledge, the first piece of related analytical work was done by Bertrand [28]. After that, Wein [29] and many related studies focus on employing the “conditional sojourn time concept” in analyzing the “due date lead time- (DDLT-)” related problems. Yano [30] develops a newsboy model to determine the “safety lead time.” Duenyas and Hopp [31] later review this problem with the semi-Markovian decision process. They also connect their work with other scheduling optimization problems. Kut and Song [32] explore quick service, quoted service time, and uniform service time systems. They develop a model which analytically captures the relationship between service time, capacity, and price. Axsäter [33] introduces a partial backorder system in which any unfilled demand can be satisfied with transshipment. Observe that in our paper, we introduce a model with the critical time point concept similar to Zhang et al.'s [34] “quoted service time,” but our model setting and optimization problem are totally different.

Inventory problems under different circumstances are usually of great complexity. An important observation from the review of the current optimal inventory control policies is that although many methods proposed in the literature are sophisticated, they are not easy to execute and implement in practice. This brings out an important aspect as argued by Alstrøm [35] that relatively few companies in practice employ the “scientifically sound” and “precisely optimal” inventory policies. In fact, the determination of optimal values for the control variables—even in a very simple inventory control system—is a complex task and therefore not favored by practitioners. Heuristic solutions are hence presented to solve (*Q*,*r*)-related inventory control problems. For instance, Yang et al. [36] propose a simple heuristic algorithm to find a near-optimal (*Q*,*r*) policy (see [37, 38] for some other related studies). In this paper, we also try to find a heuristic solution scheme which is easy to understand and to implement for practical inventory control, especially for the target healthcare centre. We derive the stocking cost, backorder cost, and lost sale cost separately and finally obtain the average cost function (of an order cycle). Since there is no simple effective method to find the analytically closed-form expression of the optimal *Q* and *r*, we develop a heuristic algorithm, which is built on an improved genetic algorithm to find the (approximately) optimal (*Q*,*r*) policy. Considering the practical situation of the healthcare centre with limited space constraint and limited throughput each day, the inventory problem cannot simply be solved by the current policies. Such kind of small or medium-sized organizations with a limited storage space which are offering a tailor-made service are not uncommon nowadays. Moreover, these organizations usually have some clients who are willing to wait for a reasonable time as they might not be able to get the ordered products immediately. Of course, they will leave if they get impatient. As for the organizations, it is necessary for them to keep the inventory in a certain level so as to keep potential customers and achieve their inventory service level target. That is why partial backlog, space capacity, service level, and waiting time control must be taken into consideration to design an appropriate “tailor-made” inventory policy feasible for such organizations to execute. However, though work has been done on inventory policies, little research has paid attention to this realistic and existing problem. Besides, few papers have conducted an analysis on the characteristics of partial backlog, space capacity, and waiting time in the field of inventory management, let alone in the healthcare relevant area. Focusing on the real-world inventory problem listed above, which is also based on the real case of the healthcare centre, it is essentially important for us to generate a customized inventory policy which contributes to the practical usage and fills the current academic gap. That is why we think the issues we consider in this paper are important. To show a clear picture about the literature positioning and originality of this paper, we prepare Table 1 which clearly outlines, item-by-item, how this paper is similar to and different from the other related studies in the literature.

## 3. The System Description

**Figure 1:**
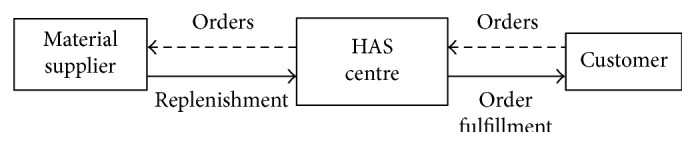
The healthcare apparel service centre supply chain system.

We concisely describe the inventory system in the context of a two-echelon make-to-order (MTO) supply chain. Consider the configuration built as a single item system driven by a Poisson demand process (see Figure 1).

**Figure 2:**
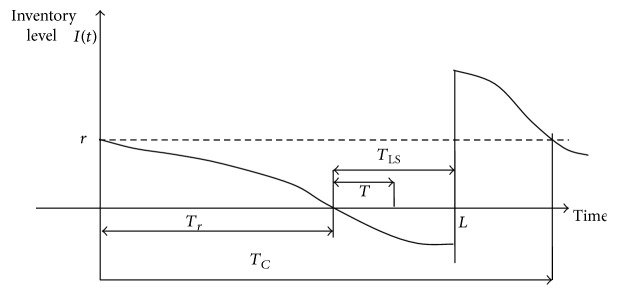
The inventory level variation in an order cycle time *T*_*C*_.

Demands are random with a mean demand rate *λ*. The inventory is reviewed continuously by a (*Q*,*r*) policy (see Figure 2): whenever the inventory position drops to *r*, the inventory manager issues an order to the supplier for a replenishment amount of *Q* units of goods, and the ordered goods arrive after a constant lead time *L*. Demand arriving at time *t* will be immediately met if the inventory level *I*(*t*) (see Figure 2) is positive. Otherwise, the customer will be told a quoted service time during which customers are willing to wait. When customers turn back after waiting, they may probably still be backordered if the orders are outstanding. Let *Ω* denote the value space of (*Q*,*r*) defined by

(1) $$\begin{array}{c} \Omega =\left\{\left(Q,r\right)\;|\;0<r<\infty ,0<Q<\infty ,r,Q\in R.\right. \end{array}$$

## 4. The Optimization Model

Consider a single-item inventory system controlled by a (*Q*,*r*) policy. Our problem is to determine the optimal inventory policy parameters *r* and *Q* to minimize the expected average cost on cycle time *C*(*Q*, *r*), where the expected average cost on a cycle time is given as follows:

(2) $$\begin{array}{c} C\left(Q,r\right)=\frac{E\left(TC\right)}{E\left[T_{C}\right]},\\ E\left[TC\right]=K+cQ+hE\left[I_{C}\right]+pE\left[LS\right]+bE\left[B_{C}\right]. \end{array}$$

*I*_*C*_ is the available stock in a cycle time and LS and *B*_*C*_ represent, respectively, the lost sales and the backorders in a cycle time.

Define

(3) $$\begin{array}{c} p\left(j;\lambda t\right)=\frac{{\left(\lambda t\right)}^{j}e^{-\lambda t}}{j!}. \end{array}$$

We have the following approximation by using the classical “integration by parts” method:

(4) $$\begin{array}{c} \int_{0}^{L}p\left(j;\lambda t\right)dt\approx \frac{1}{\lambda }P\left(j+1;\lambda L\right),\\ T_{LS}=\left\{\begin{array}{cc} 0 & T_{r}\geq L\\ L-T_{r} & T_{r}<L. \end{array}\right. \end{array}$$

where *T*_*r*_ is the time for *r* units to be depleted.

Notice that *T*_*r*_ follows an Erlang distribution with parameters *r* and *λ*, and its probability density function is *λp*(*r* − 1; *λt*). Thus, we have

(5) $$\begin{array}{c} E\left[T_{LS}\right]=\int_{0}^{L}\left(L-t\right)g\left(t\right)dt=\int_{0}^{L}\lambda \left(L-t\right)p\left(r-1;\lambda t\right)dt=LP\left(r;\lambda L\right)-\frac{r}{\lambda }P\left(r+1;\lambda L\right). \end{array}$$

The expected number of lost sales per cycle can be obtained as

(6) $$\begin{array}{c} E\left[LS\right]=\lambda E\left[T_{LS}\right]=\lambda LP\left(r;\lambda L\right)-rP\left(r+1;\lambda L\right). \end{array}$$

With the above model, we can derive Lemma 4.1.

**Lemma 1 .**

*Consider the following:*

(7) $$\begin{array}{c} E\left[T_{C}\right]=\frac{Q}{\lambda }+E\left[T_{LS}\right]. \end{array}$$

**Proof of Lemma 4.1.**

On average, all items ordered are consumed in a single cycle; the satisfied demands per unit time can be denoted as *Q*/*E*[*T*_*C*_]. It is equal to the incoming demands minus lost sales, that is,

(8) $$\begin{array}{c} \lambda -\frac{E\left[LS\right]}{E\left[T_{C}\right]}.\frac{Q}{E\left[T_{C}\right]}=\lambda -\frac{E\left[LS\right]}{E\left[T_{C}\right]},\;q.e.d. \end{array}$$

This yields Lemma 4.1.

Denote *T*_*B*_ as the time period of backorders. The existence of *T*_*B*_ is related to *T*_*r*_.

(9) $$\begin{array}{c} T_{B}=\left\{\begin{array}{cc} 0 & T_{r}>L-T\\ L-T-T_{r} & 0<T_{r}\leq L-T \end{array}\right\}. \end{array}$$

We derive the expected time period for backorders as follows:

(10) $$\begin{array}{c} E\left(T_{B}\right)=\int_{0}^{L-T}\left(L-T-t\right)g\left(t\right)dt=\int_{0}^{L-T}\left(L-T\right)g\left(t\right)dt-\int_{0}^{L-T}tg\left(t\right)dt=\left(L-T\right)P\left(r;\lambda \left(L-T\right)\right)-\frac{r}{\lambda }P\left(r+1;\lambda \left(L-T\right)\right). \end{array}$$

Thus, the expected number of backorders is given by

(11) $$\begin{array}{c} \lambda E\left(T_{B}\right)=\lambda \left(L-T\right)P\left(r;\lambda \left(L-T\right)\right)-rP\left(r+1;\lambda \left(L-T\right)\right). \end{array}$$

We can then get the cumulative backorders at time *t*, *B*(*t*), in the following:

(12) $$\begin{array}{c} B\left(t\right)=\lambda tP\left(r;\lambda t\right)-rP\left(r+1;\lambda t\right). \end{array}$$

Denote the expected backorders per cycle by *B*_*C*_. When the inventory level falls below 0, backorder costs are incurred in the time interval [*T*_*r*_, *t*]. There is no shortage penalty cost incurred by backorders during the free waiting time *T*. When *t* falls into a certain interval, *T*_*r*_ < *t* < *L* − *T*, the expression for *B*_*C*_ can be expressed as follows:

(13) $$\begin{array}{c} B_{C}=\int_{T_{r}}^{L-T}\lambda \left(t-T_{r}\right)dt. \end{array}$$

Given that the probability density function of *T*_*r*_ is *f*(*s*) = *λp*(*r* − 1; *λs*), we can find the closed-form expression for Proposition 4.2.

**Proposition 1 .**

*Consider the following:*

(14) $$\begin{array}{c} E\left[B_{C}\right]=\frac{\lambda {\left(L-T\right)}^{2}}{2}P\left(r;\lambda \left(L-T\right)\right)-\frac{\lambda \left(L-T\right)r}{\lambda }P\left(r+1;\lambda \left(L-T\right)\right)+\frac{r\left(r+1\right)}{2\lambda }P\left(r+2,\lambda \left(L-T\right)\right). \end{array}$$

**Proof of Proposition 4.2.**

Consider

(15) $$\begin{array}{c} E\left[B_{C}\right]=E\left[\int_{T_{r}}^{L-T}\lambda \left(t-T_{r}\right)dt\right]=\int_{0}^{L-T}\left[\int_{s}^{L-T}\lambda \left(t-s\right)dt\right]f\left(s\right)ds=\frac{\lambda {\left(L-T\right)}^{2}}{2}P\left(r;\lambda \left(L-T\right)\right)-\frac{\lambda \left(L-T\right)r}{\lambda }P\left(r+1;\lambda \left(L-T\right)\right)+\frac{r\left(r+1\right)}{2\lambda }P\left(r+2;\lambda \left(L-T\right)\right),\;q.e.d. \end{array}$$

Denote the expected inventory held per cycle by *E*[*I*_*C*_]. For the sake of clarity, we separate the “derivation” of *E*[*I*_*C*_] into two parts: prior to the replenishment time, denote it by *E*[*I*_*B*_], and after the replenishment time, denote it by *E*[*I*_*A*_]. Define *E*[*I*_*T*_] = *E*[*I*_*A*_] + *E*[*I*_*B*_] and *D*(*t*) as the total demand during (0, *t*]. As defined earlier, note that *I*(*t*) represents the inventory level at time *t*. We have Proposition 4.3.

**Proposition 2 .**

*Consider the following:*

(16) $$\begin{array}{c} E\left[I_{B}\right]=rL-\frac{{rL}^{2}}{2}+\frac{{rL}^{2}}{2}P\left(r-1;\lambda L\right)-rLP\left(r;\lambda L\right)+\frac{r\left(r+1\right)}{2\lambda }P\left(r+1;\lambda L\right). \end{array}$$

**Proof of Proposition 4.3.**

Consider

(17) $$\begin{array}{c} E\left[I_{B}\right]=\int_{0}^{L}E{\left[I\left(t\right)\right]}^{+}dt=\int_{0}^{L}E{\left[r-D\left(t\right)\right]}^{+}dt=\int_{0}^{L}\left\{\overset{r-1}{\underset{x=0}{\sum }}\left(r-x\right)p\left(x;\lambda t\right)\right\}dt=\overset{r-1}{\underset{j=0}{\sum }}\frac{\left(r-j\right)}{\lambda }P\left(j+1;\lambda L\right)=rL-\frac{{rL}^{2}}{2}+\frac{{rL}^{2}}{2}P\left(r-1;\lambda L\right)-rLP\left(r;\lambda L\right)+\frac{r\left(r+1\right)}{2\lambda }P\left(r+1;\lambda L\right),\;q.e.d. \end{array}$$

In the following, we deduce the expected inventory held per cycle after the replenishment time *E*[*I*_*A*_]. Let *I*(*L*) (distributed from *r* − *Q* to *r*) be the inventory level just before a replenishment order arrives and *T*(*r*) be the time for *r* demands to arrive from time 0. We have Pr{*I*(*L*) = *j*} = *p*(*r* − *j*; *λL*),  *j* = *r* − *Q*, *r* − *Q* + 1,…, 0,…, *r*, and we can derive Proposition 4.4.

**Proposition 3 .**

*Consider the following:*

(18) $$\begin{array}{c} E\left[I_{A}\right]=\frac{1}{2\lambda }\left\{\overset{r}{\underset{j=r+1-Q}{\sum }}p\left(r-j,\lambda L\right){\left(j+Q\right)}^{2}+\left(j+Q\right)-r\left(r+1\right)\right\}. \end{array}$$

**Proof of Proposition 4.4.**

Let *Z* = *Q* + *I*(*L*) be the inventory level just after a replenishment order arrives. The state space of *Z* is *r*, *r* + 1,…, *r* + *Q*. Denote *T*_*i*_ as the interarrival time of the demands. The *T_i_*, *i* = 1, 2, 3, … , are i.i.d. exponential random variables with mean 1/*λ*. The expected inventory held from the replenishment time until the next ordering time is then given by

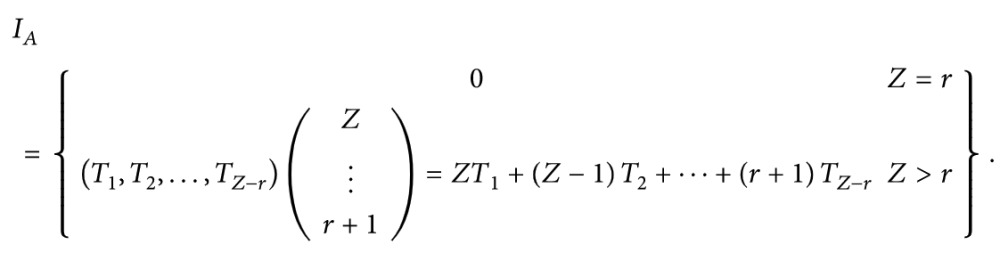
(19)

Hence, we have

(20) $$\begin{array}{c} E\left[I_{A}\right]=\frac{1}{\lambda }\overset{r+Q}{\underset{z=r+1}{\sum }}\left(\mathrm{Pr}\left(Z=z\right)\left(z+z-1+\cdots +r+1\right)\right)=\frac{1}{2\lambda }\overset{r+Q}{\underset{z=r+1}{\sum }}\left(\mathrm{Pr}\left(Z=z\right)\left(z^{2}+z-r\left(r+1\right)\right)\right)=\frac{1}{2\lambda }\left\{\overset{r+Q}{\underset{z=r+1}{\sum }}\mathrm{Pr}\left(Z=z\right)\left(z^{2}+z\right)-r\left(r+1\right)\right\}=\frac{1}{2\lambda }\left\{\overset{r}{\underset{j=r+1-Q}{\sum }}\mathrm{Pr}\left(I\left(L\right)=j\right)\left[{\left(j+Q\right)}^{2}+\left(j+Q\right)-r\left(r+1\right)\right]\right\}=\frac{1}{2\lambda }\left\{\overset{r}{\underset{j=r+1-Q}{\sum }}p\left(r-j,\lambda L\right){\left(j+Q\right)}^{2}+\left(j+Q\right)-r\left(r+1\right)\right\}. \end{array}$$

Thus,

(21) $$\begin{array}{c} E\left(I_{C}\right)=rL-\frac{{rL}^{2}}{2}+\frac{{rL}^{2}}{2}P\left(r-1;\lambda L\right)-rLP\left(r;\lambda L\right)+\frac{r\left(r+1\right)}{2\lambda }P\left(r+1;\lambda L\right)+\frac{1}{2\lambda }\left\{\overset{r}{\underset{j=r+1-Q}{\sum }}p\left(r-j,\lambda L\right){\left(j+Q\right)}^{2}+\left(j+Q\right)-r\left(r+1\right)\right\},q.e.d. \end{array}$$

In this paper, we consider the service level constraint in the inventory control model as well. In addition, if demand exceeds *r*, the inventory system will experience a shortage. Let (*X* − *r*)^+^ = max{*X* − *r*, 0}. According to Ravindran et al. [39] and Jha and Shanker [40], the expected shortage can be expressed as follows:

(22) $$\begin{array}{c} E{\left(X-r\right)}^{+}=\int_{r}^{+\infty }\left(X-r\right)dF\left(x\right)=\sigma \sqrt{L}\psi \left(r\right), \end{array}$$

where *ψ*(*r*) = *ϕ*(*r*) − *r*(1 − *Φ*(*r*)) > 0 which denotes the right linear loss function of the standard normal distribution and *ϕ*(*r*) and *Φ*(*r*) are probability density function and cumulative distribution function of the standard normal distribution, respectively.

Notice that the stockout risk target can be converted into an analytical constraint which has been considered in some inventory optimization problems in recent research (see [41, 42]). In particular, Lee et al. [42] propose an algorithm to get the “computable” order quantity for an inventory model with such a constraint. Observe further that the no-stockout risk target can be expressed as a proportion of demand that can be met from the stock in a cycle. According to Ouyang et al. [43], the no-stockout risk target constraint can be converted into the following format: $\sqrt{\lambda L}\psi \left(r\right)/Q\leq \left(1-\xi \right)\equiv n.$ Thus, the optimization problem can be expressed as the nonlinear optimization problem, Problem (P1), as shown below:

(23) $$\begin{array}{c} \underset{r,Q}{\min}C\left(Q,r\right),\\ s.t.\;r+Q\leq m,\\ \frac{\sqrt{\lambda L}\psi \left(r\right)}{Q}\leq n,\\ Q>0,\\ r>0. \end{array}$$

## 5. Data Analysis

### 5.1. Case Study

We illustrate the performance and applicability of the derived optimal inventory control model by using data collected from the healthcare apparel service centre we introduced in Section 1. Observe that the centre operates in a make-to-order (MTO) mode in which it will produce the product after the customer advises his/her specific needs with precise measurement. Thus, what the centre needs is to keep inventory of the materials and fabrics. Since the centre is a nonprofit-making charity organization, minimizing inventory costs is its major consideration (instead of profit). With the relatively small-sized inventory capacity and storage space, the centre cannot keep a lot of fabrics and materials in its stockroom. Customer orders arrive stochastically and cannot be controlled. Overall speaking, the proposed (*Q*,*r*) inventory control model is applicable to this centre.

In the following analysis, related data are collected from the healthcare centre covering the period from January to March, 2013. Without loss of generality, we set *λ* = 1, *m* = 200, and the numbers in the tables (Tables 2 [Table tab3]–4) are scaled consistently to keep the confidentiality of the sensitive data. We conduct the analysis on three separate products, namely, the wheelchair raincoat, safety jumpsuit, and apron. We consider the optimal inventory control policy for each product separately because the required fabric for each item is different and will be sourced from a different textile supplier.

Table 3 shows the parameters of the optimal policy and also the respective cost under the optimal policy.  Table 4 shows the current practice in which the ordering policy is based on the “gut feeling” of the manager. With the optimal policy, the ordering quantity of each item has a reduction (see Table 5).  Table 6 shows the improvement by using the optimal policy. From Table 6, it is obvious that the expected total cost on an order cycle is reduced substantially at around 20% with the model introduced in the paper for this specific healthcare service centre case. This is an inspiring result as the healthcare service centre can attain the same high service level while reducing the respective total cost substantially. This helps to achieve an efficient, effective, and sustainable healthcare service operations.

### 5.2. Sensitivity Analysis

**Figure 3:**
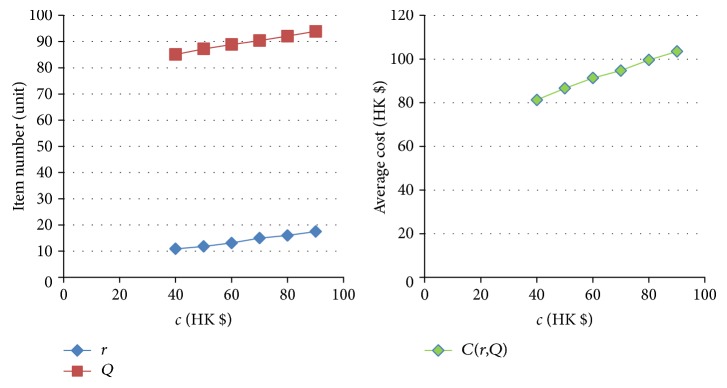
The effect of variation of *c* on *Q*, *r*, and *C(r, Q)*.

**Figure 4 fig4:**
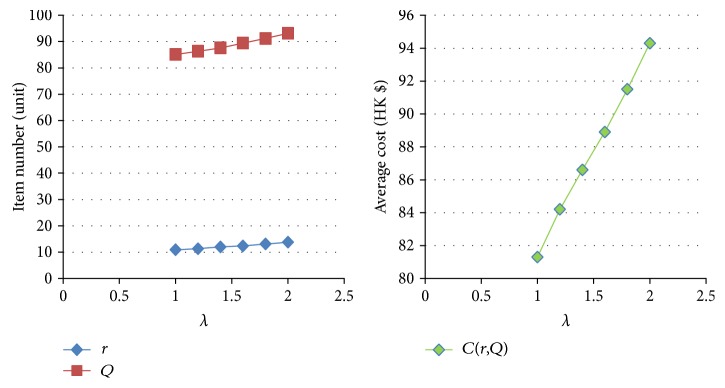
The effect of variation of *λ* on *Q*, *r*, and *C(r, Q)*.

**Figure 5 fig5:**
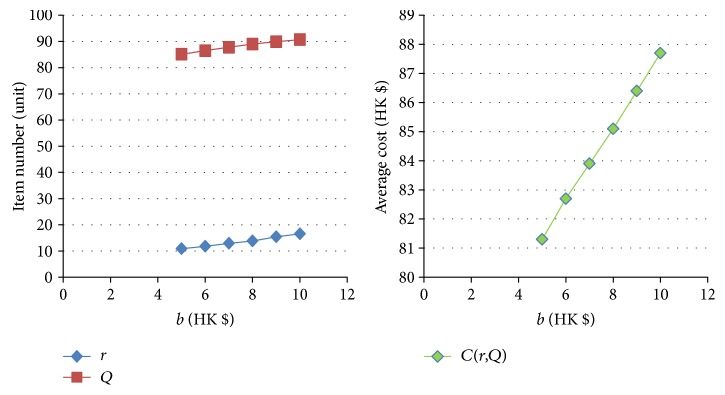
The effect of variation of *b* on *Q*, *r*, and *C(r, Q)*.

**Figure 6 fig6:**
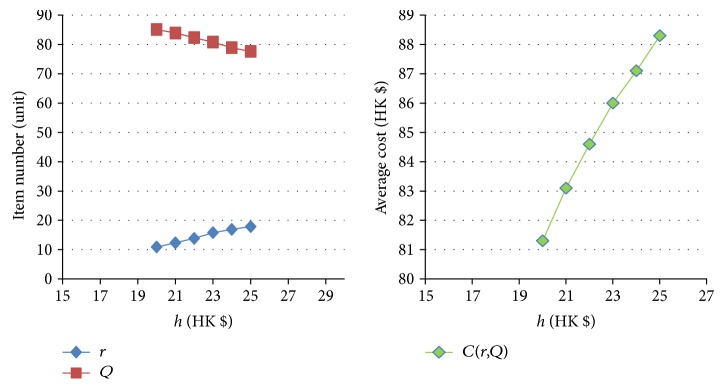
The effect of variation of *h* on *Q*, *r*, and *C(r, Q)*.

**Figure 7 fig7:**
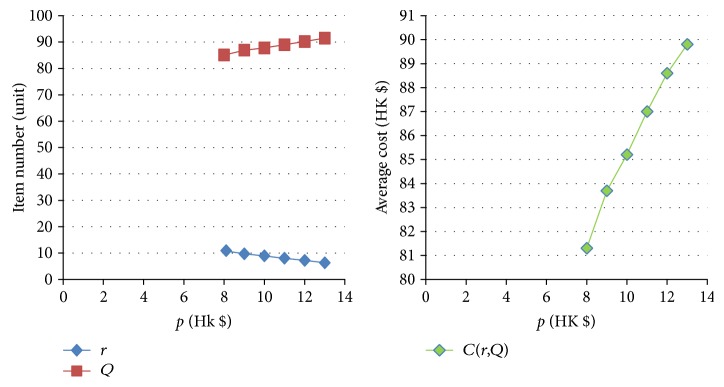
The effect of variation of *p* on *Q*, *r*, and *C(r, Q)*.

**Figure 8:**
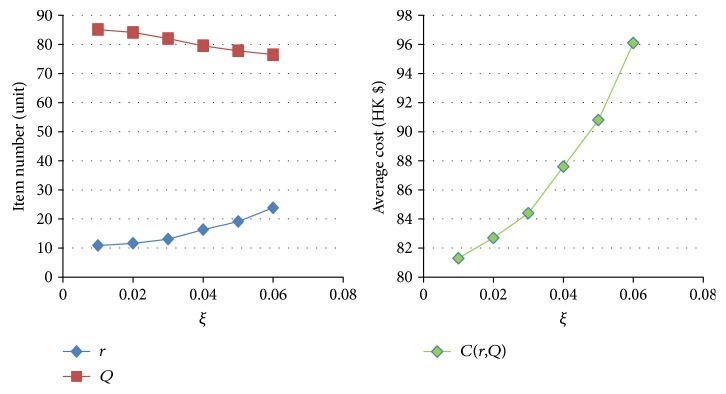
The effect of variation of *ξ* on *Q*, *r*, and *C(r, Q)*.

To generate more insights on the situation under which the optimal inventory control policy is especially efficient, sensitivity analysis is conducted on the major parameters in the optimization model. Since the three products we explored have similar features, we just present the result for the “Wheelchair Raincoat.” Tables 7 [Table tab8] [Table tab9] [Table tab10] [Table tab11]–12 show the numerical sensitivity analysis results. Figures 3 [Fig fig4] [Fig fig5] [Fig fig6] [Fig fig7]–8 clearly illustrate the effect of different parameters' variation on *r*, *Q* and *C*(*Q*, *r*). Table 13 shows a summary.

From Table 13, we can see that when the ordering (purchasing) cost *c* increases, the average inventory cost increases. This result is as expected because *c* is the purchasing cost. It is interesting to note that a larger *c* leads to a larger *Q* and a larger *r*, and this relates to the fact that a larger *Q* slows down the increasing rate of the ordering cost on each unit. For the mean demand rate *λ*, a larger *λ* leads to increases in *r*, *Q*, and cost *C*. By definition, a larger *λ* means customer demand per unit time increases. To avoid the occurrence of stockout, the optimal inventory policy will have both *r* and *Q* being larger, which also leads to a bigger average cost *C*. For the backordering cost *b*, when it increases, backorder is being penalized more, and hence both *r* and *Q* increase, which also yields a higher cost *C*. When the stockout penalty *p* increases, it brings about increases in *r* and average inventory cost. When stockout cost per unit time becomes larger, the shortage cost is magnified. To avoid the stockout situation, the level of reorder point has to be raised. For the holding cost *h*, when it becomes larger, the average inventory cost increases. It is interesting to note that the optimal *Q* derived by the nonlinear optimization model also increases. This can be explained by the fact that a larger *Q* offsets the increase in the inventory cost on each unit. Finally, when the specified service level (with respect to stockout) *ξ* increases (which means the inventory service level drops), the optimal *Q* decreases. This is intuitive as a lower quantity is needed for the case when inventory service level is lower.

### 5.3. Comparison with EOQ Model

To evaluate the significance of the proposed policy, another classical model in inventory management is used to compare with policy. Considering the background of the case study, traditional EOQ model should be extended under the conditions of partial backordering and stochastic demands. In this case, orders of size are placed from a supplier when the stock drops down to the reorder level. Due to the uncertainty in customer demand during lead time, there are chances of shortages if demand is underestimated and high holding costs if demand is overestimated. When shortages occur, they are backordered. According to Yan (2005), when an order is placed as the inventory level hits zero, the optimal order size and the minimized average total cost are derived as follows:

(24) $$\begin{array}{c} Q^{*}=\sqrt{\frac{2K\lambda }{h}},\\ C_{EOQ}=\frac{{hQ}^{*}}{2}+\frac{K\lambda }{Q}+C. \end{array}$$

Backlogging is allowed in this case, thus the optimal order size and reorder position are

(25) $$\begin{array}{c} Q^{*}=\sqrt{\frac{2K\left(p+h\right)\lambda }{hp}},\\ l^{*}=\left(\frac{h}{p+h}\right)Q^{*}. \end{array}$$

Besides, the expected average cost is

(26) $$\begin{array}{c} C\left(Q,l\right)=\left[\left(\frac{\left(p+h\right)l^{2}}{2Q}+\frac{hQ}{2}-hl\right)\pi \right]e. \end{array}$$

We have done a comparison between (*r*, *Q*) policy and EOQ policy with available data of the healthcare centre.

From the results (see Table 14), it is clear that the expected average cost per cycle time of EOQ policy is much higher than that of (*r*, *Q*) policy. We can conclude that EOQ method requires frequent replenishment with fewer order quantity of each cycle time to handle the uncertain customer demands. Back to the case study, since the healthcare centre, as a nonprofit organization, provides apparel-making service only for specific group of people, frequent replenishments might increase the ordering cost and the holding cost. In terms of these two methods, the proposed (*r*, *Q*) policy seems to be better and more appropriate for the healthcare centre to adopt.

## 6. Concluding Remarks and Future Research

Based on the real-world operations of a healthcare apparel service centre in Hong Kong, this paper explores a partial-backorder (*Q*,*r*) inventory control policy with capacity and service level constraints. An analytical optimization model is constructed to solve the problem. Employing data collected from the healthcare apparel centre, further analysis is conducted. The computational findings indicate that the expected total cost on an order cycle is reduced substantially at around 20% with the use of the optimal inventory control model introduced in the paper. This is a very remarkable result as this significant saving of the total cost would help to lead to the long-term sustainable operations of the healthcare service centre, which is a nonprofit-making service organization.

As a concluding remark, we believe that the derived inventory control model is important not only because it helps significantly improve the operations' efficiency in the specific healthcare apparel service centre as explored in this paper but it also has good implications to a more general domain: First, the service level is considered in our inventory model. For many real-world practices (including the healthcare apparel centre we mentioned in this paper), it is necessary to keep an appropriate service level so that stockout cost is controlled. Second, under our proposed policy, the expected average cost on a finite horizon is minimized so that a better budget allocation can be achieved and resources can be fully utilized. This is critical to nonprofit-making charity organizations. Third, free waiting and partial backorders are both allowed in our model. The inventory cost function with partial backordering cost is hence more comprehensive and closer to real-world practices. Fourth, we have derived various propositions on the model's structural properties which also supplement the existing literature on inventory control. Fifth, a case-based sensitivity analysis has been conducted to reveal important insights on how each major model parameter affects the optimal inventory policy and its performance (measured in cost).

Similar to other analytical studies in inventory control, our work has several limitations. For example, we currently assume that both the inventory service level and the storage space are all prespecified constraints. In future research, we plan to extend our analysis to a more challenging case when the service level and the storage space are decision variables. Considering the simplicity of operation, (*Q*,*r*) policy is adopted for the healthcare centre to solve the inventory control problem in the case study. However, other policies might also be considered to compare with (*Q*,*r*) policy. In addition, the impact of variations in multiple parameters will be analyzed in the future research.

## Figures and Tables

**Table 1:** The literature positioning of this paper.

Papers	Stochastic demand?	(*Q,r*) policy?	Heuristic solution?	Partial backlog?	Space constraint?	Waiting time?	Service level?	Healthcare related?
Federgruen and Zheng [2], Forsberg [17]	Yes	Yes	Yes	No	No	No	No	No
Silver [8], Goyal and Satir [6], Browne and Zipkin [4], Eynan and Krop [5], Tarim and Kingsman [9], Ben-Daya and Noman [3], He et al. [10], Li et al. [11]	Yes	No	No	No	No	No	No	No
Lau and Lau [7]	Yes	No	No	No	Yes	No	No	No
Forsberg [17], Zheng [44], Lau et al. [45], Song et al. [12], Berk and Gürler [13], Axäter [14], Axäter [15], Axäter [16]	Yes	Yes	No	No	No	No	No	No
Montgomery et al. [23]	Yes	No	Yes	Yes	No	No	No	No
Kim and Park [24]	Yes	Yes	No	Yes	No	Yes	No	No
Moinzadeh [25]	No	No	No	Yes	No	Yes	No	No
Rabinowitz et al. [26]	Yes	Yes	No	Yes	No	No	No	No
Hu et al. [27]	No	No	No	Yes	No	Yes	No	No
Bertrand [28], Wein [29], Yano [30], Duenyas and Hopp [31], Kut and Song [32]	No	No	No	No	No	Yes	No	No
Axsäter [33]	Yes	No	No	Yes	No	Yes	No	No
Zhang et al. [34]	No	Yes	No	Yes	No	Yes	No	No
Platt et al. [38], Gallego [37], Yang et al. [36]	No	Yes	Yes	No	No	No	No	No
Jha and Shanker [40], Chu et al. [41], Lee et al. [42], Ouyang et al. [43]	No	No	No	No	No	No	Yes	No
Buzacott and Shanthikumar [18], Wang et al. [22]	No	No	No	No	No	Yes	No	No
Özer and Wei [21]	No	No	No	No	Yes	Yes	No	No
This paper	Yes	Yes	Yes	Yes	Yes	Yes	Yes	Yes

**Table 2:** Parameters of the model (per one unit of the item).

	*c*	*h*	*p*	*b*	*L*	*α*	*ξ*
Wheelchair raincoat	40	20	8	5	3	1	0.01
Safety jumpsuit	45	24	9	6	5	0.95	0.015
Apron	42	22	8	3	2	0.85	0.01

**Table 3 tab3:** Optimal policy of *r* and *Q* in service level constraint.

	(*r*, *Q*)	*C*(*r*, *Q*)
Wheelchair raincoat	(10.90,85.1)	81.3
Safety jumpsuit	(8.72,77.3)	74.7
Apron	(7.98,71.5)	61.9

**Table 4:** Current practice (in the centre).

	(*r*, *Q*)	*C*(*r*, *Q*)
Wheelchair raincoat	(18,90)	107.3
Safety jumpsuit	(13,80)	93.1
Apron	(10,75)	78.8

**Table 5:** Reduction of batch size by using the optimal policy.

	Reduction on *Q*	Percentage reduction on *Q*
Wheelchair raincoat	5.3	5.89%
Safety jumpsuit	2.7	3.38%
Apron	3.5	4.67%

**Table 6:** Inventory cost improvement by using the optimal policy.

	Cost saving	Percentage cost saving
Wheelchair raincoat	25.4	23.7%
Safety jumpsuit	17.6	18.9%
Apron	16.7	21.2%

**Table 7:** The effect of variation of *c* on *Q* and *r.*

*c*	(*r*, *Q*)	*C*(*r*, *Q*)
40	(10.90,85.1)	81.3
50	(11.81,87.2)	86.6
60	(13.12,88.9)	91.3
70	(14.98,90.4)	94.7
80	(16.01,92.1)	99.6
90	(17.47,93.9)	103.5

**Table 8 tab8:** The effect of variation of *λ* on *Q* and *r.*

*λ*	(*r*, *Q*)	*C*(*r*, *Q*)
1	(10.90,85.1)	81.3
1.2	(11.32,86.3)	84.2
1.4	(11.98,87.6)	86.6
1.6	(12.36,89.4)	88.9
1.8	(13.07,91.2)	91.5
2.0	(13.78,93.1)	94.3

**Table 9 tab9:** The effect of variation of *b* on *Q* and *r.*

*b*	(*r*, *Q*)	*C*(*r*, *Q*)
5	(10.90,85.1)	81.3
6	(11.82,86.5)	82.7
7	(12.93,87.8)	83.9
8	(13.83,89.0)	85.1
9	(15.42,89.9)	86.4
10	(16.56,90.7)	87.7

**Table 10 tab10:** The effect of variation of *h* on *Q* and *r.*

*h*	(*r*, *Q*)	*C*(*r*, *Q*)
20	(10.90,85.1)	81.3
21	(12.37,83.9)	83.1
22	(13.85,82.3)	84.6
23	(15.81,80.8)	86.0
24	(16.92,78.9)	87.1
25	(17.88,77.6)	88.3

**Table 11 tab11:** The effect of variation of *p* on *Q* and *r.*

*p*	(*r*, *Q*)	*C*(*r*, *Q*)
8	(10.90,85.1)	81.3
9	(9.72,86.9)	83.7
10	(8.91,87.8)	85.2
11	(8.02,89.0)	87.0
12	(7.23,90.2)	88.6
13	(6.31,91.5)	89.8

**Table 12:** The effect of variation of *ξ* on *Q* and *r.*

*ξ*	(*r*, *Q*)	*C*(*r*, *Q*)
0.01	(10.90,85.1)	81.3
0.02	(11.65,84.1)	82.7
0.03	(13.08,82.0)	84.4
0.04	(16.35,79.5)	87.6
0.05	(19.12,77.8)	90.8
0.06	(23.87,76.5)	96.1

**Table 13:** Summary of sensitivity analysis.

Parameter	Effect on *r*	Effect on *Q*	Effect on cost *C*
Purchasing cost *c ↑*	*↑*	*↑*	*↑*
Mean demand rate *λ ↑*	*↑*	*↑*	*↑*
Backorder cost *b ↑*	*↑*	*↑*	*↑*
Stockout cost *p ↑*	*↑*	↓	*↑*
Holding cost *h ↑*	↓	*↑*	*↑*
Specified service level *ξ ↑*	*↑*	↓	*↑*

**Table 14:** Comparison between proposed (*r*, *Q*) policy and EOQ model.

	*Q*	Expected average cost/cycle time
(*r*, *Q*) policy	85.1	81.3
EOQ policy	18.7	167.76

## Parameters

*T*_*C*_: The order cycle time
*T*: The service time
*T*_*r*_: The time for *r* units to be depleted
*T*_LS_: The time period in which lost sales occur
*K*: The fixed ordering cost
*c*: The purchasing cost per unit
*p*: The stockout risk associated cost (per unit stockout)
*h*: The holding cost per unit per time unit
*b*: The backordering cost per unit per time unit
*X*: The stock level (continuous random variable)
*I*_*C*_: The expected stock in a cycle
*I*(*t*): The inventory level at time *t*
*g*(*t*): The probability density function of time *t*
LS: The number of unfulfilled demand, just like “lost sale”
*B*_*C*_: The backorders in a cycle
*k*: The safety factor
*m*: The maximum capacity
*ξ*: The target stockout risk tolerance level
*n*: 1 − *ξ* (the target no-stockout risk level)
*λ*: The demand rate
*L*: The lead time
*Q*: The order quantity (as a decision variable).
